# Preconditioning of Mesenchymal Stem Cells Enhances the Neuroprotective Effects of Their Conditioned Medium in an Alzheimer’s Disease In Vitro Model

**DOI:** 10.3390/biomedicines12102243

**Published:** 2024-10-02

**Authors:** Tatiana Tolstova, Ekaterina Dotsenko, Natalia Luzgina, Alexander Rusanov

**Affiliations:** Institute of Biomedical Chemistry, Pogodinskaya 10, 119121 Moscow, Russia

**Keywords:** Alzheimer’s disease, MSCs, secretome, PC12, neuroprotection, microglia, immunosuppression

## Abstract

Background: Alzheimer’s disease (AD) develops as a result of oxidative damage to neurons and chronic inflammation of microglia. These processes can be influenced by the use of a conditioned medium (CM) derived from mesenchymal stem cells (MSCs). The CM contains a wide range of factors that have neurotrophic, antioxidant, and anti-inflammatory effects. In addition, the therapeutic potential of the CM can be further enhanced by pretreating the MSCs to increase their paracrine activity. The current study aimed to investigate the neuroprotective effects of CM derived from MSCs, which were either activated by a TLR3 ligand or exposed to CoCl_2_, a hypoxia mimetic (pCM or hCM, respectively), in an in vitro model of AD. Methods: We have developed a novel in vitro model of AD that allows us to investigate the neuroprotective and anti-inflammatory effects of MSCs on induced neurodegeneration in the PC12 cell line and the activation of microglia using THP-1 cells. Results: This study demonstrates for the first time that pCM and hCM exhibit more pronounced immunosuppressive effects on proinflammatory M1 macrophages compared to CM derived from untreated MSCs (cCM). This may help prevent the development of neuroinflammation by balancing the M1 and M2 microglial phenotypes via the decreased secretion of proinflammatory cytokines (IL-1β, IL-6, and TNF-α) and increased secretion of IL-4, as well as the expression of *IL-10* and *TGF-β* by macrophages. Moreover, a previously unknown increase in the neurotrophic properties of hCM was discovered, which led to an increase in the viability of neuron-like PC12 cells under H_2_O_2_-induced oxidative-stress conditions. These results are likely associated with an increase in the production of growth factors, including vascular endothelial growth factor (VEGF). In addition, the neuroprotective effects of CM from preconditioned MSCs are also mediated by the activation of the Nrf2/ARE pathway in PC12 cells. Conclusions: TLR3 activation in MSCs leads to more potent immunosuppressive effects of the CM against pro-inflammatory M1 macrophages, while the use of hCM led to increased neurotrophic effects after H_2_O_2_-induced damage to neuronal cells. These results are of interest for the potential treatment of AD with CM from preactivated MSCs.

## 1. Introduction

Alzheimer’s disease (AD) is a progressive neurodegenerative disorder that causes cognitive impairment and dementia. It is caused by a variety of destructive processes, such as the loss of cholinergic neurons, the formation of neurofibrillary tangles (NFTs) and β-amyloid (Aβ)-containing senile plaques, glial cell activation, and inflammation [1]. Oxidative stress plays a significant role in the development of AD by contributing to these mechanisms and combining them [2].

A promising strategy for the treatment of inflammatory and neurodegenerative diseases, such as AD, is the use of mesenchymal stromal cells (MSCs). These cells have immunomodulatory and regenerative properties, making them valuable tools for medical research. It is known that MSCs have an effect on the pathogenesis of Alzheimer’s disease, including the formation of NFTs and Aβ-containing senile plaques. These effects are primarily associated with the influence of MSCs on neuroinflammation and microglia [3]. However, the specific mechanisms behind these effects remain to be fully understood. Despite this, studies have shown that MSC transplantation can improve amyloid-beta pathology by altering the function of microglia and reducing oxidative stress [4]. Additionally, MSCs can enhance autophagy, which leads to increased clearance of amyloid-beta [5]. Furthermore, MSCs may also be able to modulate the formation of NFTs through their influence on mitochondrial dysfunction in neurons [6]. The use of conditioned media (CM) from MSCs has also been reported as a promising strategy for the treatment of AD. An MSC-derived conditioned medium can activate the Keap1-Nrf2-HO-1 antioxidant defense pathway in cells [7,8], reducing the level of intracellular reactive oxygen species (ROS) [9]. This process protects neurons from apoptosis by inhibiting caspase-3 and Bax and activating the antiapoptotic protein Bcl-2 [9,10]. In addition, MSCs produce neuroregulatory molecules, such as nerve growth factor (NGF), brain-derived neurotrophic factor (BDNF), and glial cell-derived neurotrophic factor (GDNF) [11,12,13]. Recent studies have also shown that the neuroprotective effects of MSC-derived CM are mediated through the activity of immunosuppressive cytokines [12,14,15,16], as well as interleukin (IL)-6 and vascular endothelial growth factor (VEGF), which support cell viability and neuronal growth [17,18]. In particular, increased secretion of anti-inflammatory factors such as IL-4, IL-10, and transforming growth factor beta (TGF-β) by MSCs leads to a reduction in the inflammatory activation of microglia, which are involved in AD pathogenesis [14,15,19]. Strategies to modulate, maintain, and enhance the paracrine activity of MSCs are being actively investigated to develop enriched MSC populations with enhanced therapeutic potential.

Inflammatory preconditioning is a technique used to increase the secretory activity and immunomodulatory properties of MSCs. This approach may also hold promise in the development of therapeutic strategies for Alzheimer’s treatment, aimed at enhancing the effects of MSC-derived CM. Several studies have confirmed the efficacy of using CMs from primed cells in in vitro models for various chronic inflammatory diseases. Thus, in a study conducted by Giannasi et al., the levels of anti-inflammatory factors (TGF-β, IL-4, and IL-10) and growth factors (hepatocyte growth factor (HGF), VEGF, BDNF, and fibroblast growth factor 2 (FGF2)) were increased in CM from MSCs pretreated with TNF-α and IL-1β. This preconditioning approach enhanced the efficacy of CM in an osteoarthritis model, reducing the activity of matrix metalloproteinases (MMPs) [16]. In addition, exosomes derived from MSCs pretreated with TNF-α promote the survival and neurogenesis of human retinal ganglion cells, likely through increased VEGF secretion [20]. Preconditioning with TNF-α also leads to enhanced secretion of the important neurotrophic factor BDNF [16,20]. The application of CM from IFN-γ-treated MSCs has been shown to increase cell viability, reduce IL-1β secretion, and decrease the level of intracellular ROS production in a cellular model of acute human lung injury [21]. The activation of Toll-like receptor 3 (TLR3) in MSCs is known to reduce the heterogeneity of the cell population and enhance their immunomodulatory properties [22,23]. We previously demonstrated that preconditioning MSCs with the TLR3 ligand polyinosinic:polycytidylic acid (poly(I:C)) improves their ability to suppress the activation and proliferation of model T cells and increases the expression of immunosuppressive molecules, such as *IDO1*, *TNFAIP6*, and *PTGES2* [22], as well as the secretion of IL-6 [24].

Hypoxic preconditioning is another promising strategy to increase the paracrine activity of MSCs. Zhang et al. demonstrated the therapeutic potential of CM from hypoxic preconditioned MSCs in an in vitro ischemia model. The use of CM resulted in an increase in neuronal viability under oxygen- and glucose-deprivation conditions, as well as the preservation of formed neurites through a VEGF-dependent mechanism [25]. Furthermore, the administration of CM derived from hypoxia-preconditioned MSCs has been shown to have a positive effect on an in vivo model of Alzheimer’s disease induced by Aβ. Specifically, CM treatment has contributed to increased neurogenesis, a reduced rate of Aβ deposition, and decreased levels of TNF-α and IL-1β in the rat hippocampus [26]. Moreover, hypoxia mimetics such as cobalt chloride (CoCl_2_), deferoxamine (DFO), and 2,4-dinitrophenol (DNP) are conveniently used to simulate hypoxic conditions in experimental models [27]. This allows some of the difficulties associated with the use of hypoxic chambers, such as unstable oxygen concentrations, to be overcome. In a study by Isildar et al., CM from DFO-treated MSCs contained higher levels of IL-4, IL-10, IL-17, and IFN-γ than did CM from untreated MSCs. In an in vivo model of type 1 diabetes, the use of CM derived from DFO-treated MSCs led to an increase in the population of regulatory T cells (Tregs) and a decrease in proinflammatory cytokines [28]. At the same time, preconditioning MSCs with CoCl_2_ mimics hypoxia, leading to increased levels of hypoxia-inducible factor 1α (HIF-1α) and VEGF [29,30,31], which play important roles in the neuroprotective effects of MSCs. However, the effect of cobalt chloride on the neuroprotective properties of CM from MSCs has not been investigated previously.

In this study, a cellular model of AD was used to investigate the potential neuroprotective effects of MSC-derived CM. Specifically, the PC12 cell line, a rat adrenal pheochromocytoma, is commonly used in in vitro studies on AD pathogenesis because of its ability to mimic the characteristics of neurons through NGF-induced differentiation [32,33]. PC12 cells are able to synthesize, store, and release norepinephrine and dopamine, compared to other cell models. Additionally, neurotransmitter receptors associated with AD, such as N-methyl-D-aspartate (NMDAR) and cholinergic receptors, are present on the surface of PC12 cells. Specifically, the expression of the NR1 and NR2 subunits of these receptors can be observed in PC12 cells [33]. The formation of ROS can be induced by exposing the cells to H_2_O_2_, mimicking the neurotoxic damage associated with AD. As microglial inflammation has also been linked to the development of AD, the THP-1 cell line differentiated into macrophages can be used to investigate the immunomodulatory effects of MSC-derived CMs [32,34,35,36].

We hypothesized that CM derived from MSCs with activated TLR3 (pCM) or pretreated with a hypoxia mimetic (hCM) might have greater neuroprotective and/or immunosuppressive effects than CM from untreated MSCs (cCM). Therefore, the aim of this study was to investigate the neuroprotective properties of the secretome of preconditioned MSCs in an experimental in vitro model of Alzheimer’s disease.

## 2. Materials and Methods

### 2.1. PC12 Cell Cultivation

Rat adrenal pheochromocytoma PC12 cells were kindly provided by Dr. Mikhail Akimov (Shemyakin–Ovchinnikov Institute of Bioorganic Chemistry, Russian Academy of Sciences). PC12 cells were cultured in RPMI-1640 medium (PanEco, Moscow, Russia) supplemented with 10% fetal bovine serum (FBS), 5% horse serum (HS), 1% GlutaMAX™, and penicillin/streptomycin (100 UI/mL and 100 μg/mL) (all from Gibco, Grand Island, NY, USA) in a humidified incubator (SANYO, Tokyo, Japan) at 37 °C, with 5% CO_2_. The cells were treated with 0.25% trypsin (PanEco, Russia) to harvest from the culture plastic surface and seeded at a density of 9 × 10⁵ cells/cm^2^ per 60 mm Petri dish (Corning Inc., Corning, NY, USA). The PC12 cells were reseeded every 3–4 days, and no more than 15 passages were used.

### 2.2. PC12 Cell Differentiation into Neuron-like Cells

PC12 cells were cultured in 6-well plates (20 × 10^4^ cells/well) or 96-well plates (2.5 × 10^4^ cells/well) for 7 days in induction medium (IM). The IM for PC12 cell differentiation consisted of RPMI-1640 (PanEco, Russia) with a reduced HS concentration (up to 1%) and 100 ng/mL NGF (Sigma-Aldrich, St. Louis, MO, USA). The IM was changed every 72 h.

The effectiveness of PC12 cell differentiation was evaluated by counting the number of neuron-like cells (with neurites) and measuring the length of the formed neurites after adding IM via an inverted microscope (Carl Zeiss, Jena, Germany). Microphotographs were taken on the 1st (as a control), 3rd, and 7th days of differentiation induction and then processed via ImageJ software (version 2.3.0/1.53q) with the NeuronJ plug-in. The cells with neurites were counted, and the lengths of the neurites were measured in at least 100 cells in three independent fields in each image. The percentage of neuron-like cells was calculated by dividing the number of cells with neurites by the total number of cells and multiplying by 100%.

### 2.3. Protocol of H_2_O_2_ Treatment

To induce Alzheimer-like neuronal injury, differentiated (neuron-like) PC12 cells (pre-seeded at 2.5 × 10^4^ cells/well) were exposed to H_2_O_2_ (trPC12). To assess the neurotoxicity of H_2_O_2_, we measured the number of viable trPC12 cells via an MTT assay 2 h after exposure to different concentrations of H_2_O_2_, ranging from 50 to 800 μM. To investigate changes in the intracellular ROS level, activation of ROS-related genes, and induction of apoptosis, we treated PC12 cells with H_2_O_2_ at a concentration of 200 μM for 2 h.

### 2.4. THP-1 Cell Cultivation and Differentiation into M0 Macrophages

The human leukemic cell line THP-1 (the shared research facility “Vertebrate Cell Culture Collection”, Russia) was cultured in RPMI-1640 medium (PanEco, Moscow, Russia) supplemented with 10% FBS, 1% GlutaMAX^TM^, and penicillin/streptomycin (100 UI/mL and 100 μg/mL) (all from Gibco, Waltham, MA, USA) at 37 °C and 5% CO_2_. The cells were seeded at a density of 5 × 10^5^ cells/mL into 25 cm^2^ culture flasks for tissue cultivation (Corning Inc., Corning, NY, USA) and passaged every 3 days. The THP-1 cells were cultured until the 15th passage.

THP-1 cells were differentiated into M0 macrophages using 10 ng/mL phorbol 12-myristate-13-acetate (PMA) (Sigma-Aldrich, Burlington, MA, USA) for 24 h. The efficiency of the cell differentiation process was confirmed by examining the cell morphology via an inverted microscope (Carl Zeiss, Oberkochen, Germany).

### 2.5. MSC Expansion

The total pool (three individual donors) of human adipose-derived MSCs was purchased from the cryobank of the Perspectiva Research and Production Company (Novosibirsk, Russia).

MSCs were cultured in culture medium α-MEM (PanEco, Russia) supplemented with 10% FBS, 1% GlutaMAX™, and penicillin/streptomycin (100 UI/mL and 100 μg/mL) (all from Gibco, USA) at 37 °C and 5% CO_2_. The cells were trypsinized at 70–80% confluence via 0.25% trypsin solution (PanEco, Russia). The cells were then seeded at 5–7 × 10^3^ cells/cm^2^ into 75 cm^2^ tissue culture flasks (Corning Inc., USA). The cells were used up to passage five.

### 2.6. MSC Differentiation

To differentiate MSCs, complete culture medium α-MEM (PanEco, Russia) supplemented with osteogenic or adipogenic inducers was used (Table 1). The medium was changed every 72 h.

To confirm their osteogenic and adipogenic potential, MSCs in the induced state were stained with Alizarin Red and Oil Red O (Sigma-Aldrich, USA) on the 14th day of cultivation by phase-contrast microscopic observation (a Primovert microscope, Carl Zeiss). Alizarin Red was used to detect the calcium deposits that are characteristic of osteocytes, whereas Oil Red O was used to identify lipids in the cytosol of adipocytes.

### 2.7. Immunophenotype Characterization of MSCs

MSCs were verified for positive staining for CD105 (Cloud-Clone Corp., Wuhan, China), CD90 (Cloud-Clone Corp., China) and CD73 (Cloud-Clone Corp., China) and negative staining for CD45 (Abcam, Cambridge, UK), CD34 (Abcam, UK), and HLA-DR (Thermo Fisher Scientific, Waltham, MA, USA). For this purpose, MSCs (5 × 10^5^ cells) were incubated with appropriate monoclonal antibodies and isotype control antibodies (Bio-Rad, Hercules, CA, USA), according to the manufacturer’s recommendations. After that, the cells were washed with PBS by centrifugation (300× *g*, 5 min), resuspended in PBS, and analyzed via a ZE5 Cell Analyzer (Bio-Rad, USA). Floreada.io software was used for data analysis (https://floreada.io/analysis, assessed on 1 December 2023, the last update was carried out in August 2024).

### 2.8. MSCs Priming Protocols

To activate TLR3, MSCs were seeded at a density of 5–7 × 10^3^ cells/cm^2^. After 24 h, the medium was replaced with fresh medium containing the TLR3 agonist. Poly(I:C) (Sigma-Aldrich, USA) was used as a TLR3 ligand at a concentration of 10 μg/mL for 3 h, as previously described [22].

MSCs were seeded at the same density (5–7 × 10^3^ cells/cm^2^) for hypoxia induction. After 24 h, 100 μM CoCl_2_·6H_2_O, a hypoxia mimetic agent (Sigma-Aldrich, USA), was added to the culture medium, and the mixture was incubated for 3 h.

After treatment with poly(I:C) or hypoxia mimetic, the MSCs were washed three times with PBS (PanEco, Russia) and incubated in RPMI-1640 medium (PanEco, Russia) without FBS/HS for 3 or 24 h for qRT‒PCR or to collect conditioned medium for ELISA, respectively.

### 2.9. Collection of MSC-Derived CM

Conditioned media derived from intact MSCs (cCM), MSCs with activated TLR3 (pCM), and hypoxia mimetic-preconditioned MSCs (hCM) were concentrated via Amicon Ultra15 MWCO filters with 3 kDa pores (Millipore, Merck KGaA, Darmstadt, Germany) at 4000× *g* for 45 min. After that, aliquots of concentrated conditioned media were stored at −86 °C. The protein concentration was measured via a bicinchoninic acid protein assay (BCA assay, Elabscience, Wuhan, China), according to the manufacturer’s recommendations. The protein concentration in CMs was confirmed to be 10-fold greater than the actual protein concentration in culture media before concentration.

### 2.10. Treatment of the PC12 Cell Line with CM

Collected and concentrated CMs (cCM, pCM, and hCM) were added to the PC12 cell line (undifferentiated or differentiated) at a 50/50 ratio by volume for a 24-hour incubation to evaluate the neuroprotective properties of the MSCs [18,37].

### 2.11. Evaluation of CM Neurotrophic Potential

PC12 cells were treated with CM, as described previously (Section 2.10), to evaluate the neurogenic potential of CM. The complete culture medium was used as a control (without NGF). The cell morphology was examined on days 1, 3, and 7 of cultivation in the presence of CMs via an inverted microscope (Carl Zeiss, Germany), as described in Section 2.2. The micrographs were processed via ImageJ software with the NeuronJ plugin.

### 2.12. Investigation of the Neuroprotective Properties of CM (MTT Assay)

The viability of trPC12 cells was evaluated via the MTT assay. After the culture medium was removed, 100 μL of MTT solution (1 mg/mL, Sigma-Aldrich, USA) was added to each well of a 96-well plate containing trPC12 cells, and the cells were incubated for 2 h. The formed formazan crystals were then dissolved in dimethyl sulfoxide (DMSO, Helicon, Moscow, Russia) by adding 100 μL/well. The absorbance of the formazan solution was measured at 570 nm via an iMark microplate reader (Bio-Rad, USA). To determine the number of viable cells, the optical density (OD) values obtained were normalized to the OD values of control samples from the same CM and expressed as percentages.

### 2.13. Assessment of ROS Levels in trPC12 Cells

The formation of intracellular ROS in trPC12 cells was assessed via the fluorescent dye chloromethyl derivative of 2′,7′-dichlorodihydrofluorescein diacetate (CM-H_2_DCFDA) (Invitrogen, Carlsbad, CA, USA), according to the manufacturer’s instructions. In the presence of ROS, CM-H_2_DCFDA undergoes oxidation, resulting in the formation of the fluorescent compound dichlorofluorescein (DCF). To normalize the number of DCF-positive cells, Hoechst-33342 staining was also used (Thermo Fisher Scientific, USA).

Thus, differentiated PC12 cells (dPC12) were treated with CM to activate the signaling pathways required for ROS neutralization, and 200 µM H_2_O_2_ was added. After 2 h of H_2_O_2_ exposure, dead cells that had detached from the bottom of the culture well were removed, and the live cells were incubated with CM-H_2_DCFDA and Hoechst-33342 for 0.5 h. The level of fluorescence in the wells was detected via a ZOE Fluorescent Cell Imaging System (Bio-Rad, USA). The mean fluorescence intensity was calculated via ImageJ software and normalized to that of the control (PC12 cells incubated without H_2_O_2_). The fluorescent cells were counted from at least 100 cells from three independent fields of each image. The number of intracellular ROS (ROS level, %) was expressed as a percentage of DCF-stained cells out of the total number of Hoechst-33342-stained live cells.

### 2.14. Effect of CM on M1/M2 Phenotypes Macrophage Differentiation

To study M1-like macrophages, M0 macrophages, obtained as described in Section 2.4, were treated with lipopolysaccharide (LPS; Thermo Fisher Scientific, USA) at a concentration of 1 μg/mL for 72 h [32,34,35]. The immunomodulatory potential of CM was evaluated by adding cCM, pCM, or hCM to M0 macrophages simultaneously with LPS. All types of CMs (cCM, pCM, and hCM) were used to differentiate M0 macrophages into M2 macrophages. Conditioned media were added to M0 cells at a 50/50 volume ratio. Polarized M0, M1, and M2 macrophages and conditioned media were collected after 72 h of incubation with CM for qRT‒PCR and ELISA, respectively.

### 2.15. qRT‒PCR

RNA was isolated from the MSC, PC12, and THP-1 cell lines via the RNeasy Mini Kit (Qiagen, Hilden, Germany), according to the manufacturer’s instructions. The concentration and purity of the RNA were determined via a nanodrop spectrophotometer (Thermo Fisher Scientific, USA). Reverse transcription was carried out via an MMLV RT kit (Evrogen, Moscow, Russia). qRT‒PCR was performed via a qPCRmix-HS SYBR+LowROX kit (Evrogen, Russia) on a CFX96 Real-Time PCR Detection System (Bio-Rad Laboratories, USA). The primer sequences are provided in Table 2. Data analysis was performed via the ΔΔCt method, with *ACTB* and/or *GAPDH* used as housekeeping genes. The relative expression of *IDO1*, *TNFAIP6*, *PTGES2*, *MRC1*, *IL10*, *TGFB1*, *HMOX1*, *NQO1*, and *BCL2* was assessed in this study. The raw data were processed via CFX Maestro 1.0 software version 4.0.0325.0418.

### 2.16. ELISA

Cytokines in cCM, pCM, hCM, and CM collected after the treatment of M0, M1, and M2 macrophages were analyzed via ELISA kits for interleukin-1β, interleukin-4, interleukin-6, TNF-α, and VEGF (all from VectorBest, Novosibirsk, Russia) in accordance with the manufacturer’s instructions. The optical density was measured at 450 nm via an absorbance reader on iMark microtiter plates (Bio-Rad, USA).

### 2.17. Statistical Analysis

The data are presented for three repeated samples and experiments, unless otherwise indicated. The results are presented as the mean ± standard error of the mean (SEM). Differences between the experimental groups were assessed via one-way analysis of variance (ANOVA), with Tukey’s post hoc test or two-way ANOVA with Sidak’s multiple comparisons. Statistical significance was determined at *p* < 0.05. GraphPad Prism version 10.1.1 was used for data analysis and plotting. The groups that were compared are marked by lines.

## 3. Results

### 3.1. Differentiation of PC12 and THP-1 Cell Lines

**Figure 1:**
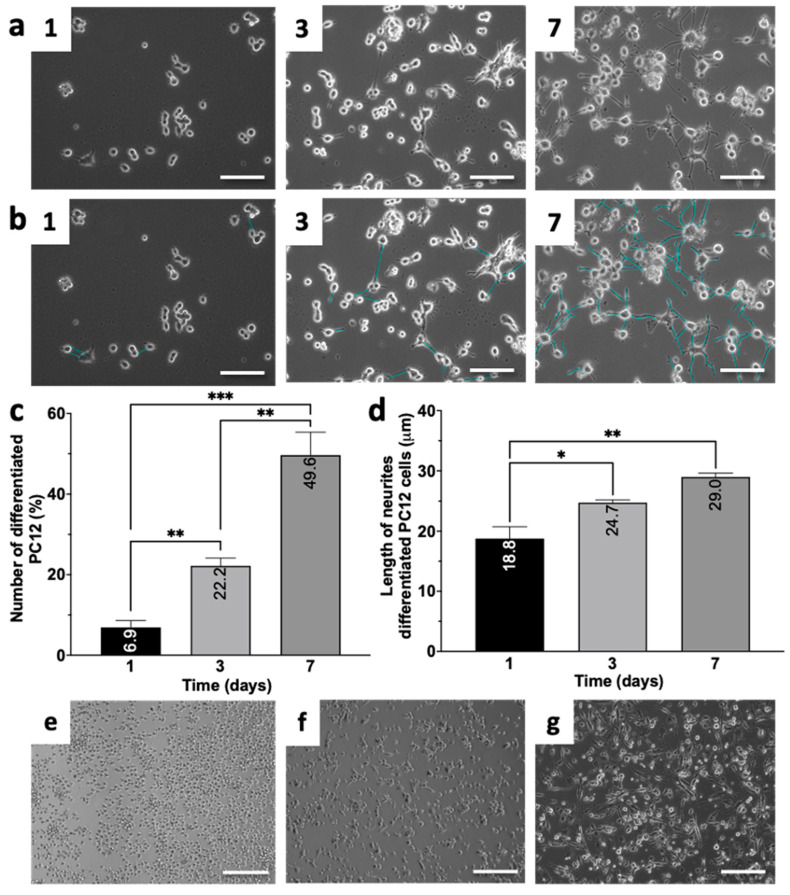
NGF-induced neuronal differentiation of PC12 cells and the PMA-induced differentiation of THP-1 cells into macrophages. Investigation of morphology (**a**), neurite length (**b**,**d**), and relative number of differentiated PC12 cells (**c**). The neurites are highlighted in blue (**b**). Micrographs of THP-1 cells without PMA treatment (**e**), after PMA treatment (**f**) (10 ng/mL, 24 h), and after LPS-induced differentiation into the M1 phenotype (**g**) (10 ng/mL PMA, 24 h + 1 μg/mL LPS, 72 h). Scale bars, 50 μm (**a**,**b**) or 100 μm (**e**–**g**). The error is indicated as SEM. * *p* < 0.05, ** *p* < 0.01, and *** *p* < 0.001.

NGF induces the differentiation of the rat adrenal pheochromocytoma PC12 cell line from replicating chromaffin-like cells into a nonreplicating population of sympathetic neuron-like cells [33]. In this study, PC12 cells were differentiated via NGF (Figure 1). The effectiveness of differentiation was assessed by the number of neuron-like cells, i.e., by measuring the number of neurites (Figure 1a) and the length of the formed neurites (Figure 1b). The cells on the 1st day of induction were considered as a control.

It has been shown that the differentiation of PC12 cells correlates with the duration of NGF induction. According to the results of the microphotography analysis, the percentages of differentiated PC12 cells on the 3rd and 7th days of NGF induction were 22.2 ± 3.4% and 49.6 ± 9.8%, respectively (Figure 1c). Additionally, measurements of neurite length revealed an increase from 18.8 ± 3.4 to 24.7 ± 0.8 μm by the 3rd day and to 29.0 ± 1.1 μm by the 7th day of induction (Figure 1d).

THP-1 cells were differentiated into M0 macrophages to be used as a microglial component in an Alzheimer’s disease model [32]. The morphology of the THP-1 cells before and after PMA-induced differentiation is shown in Figure 1e,f. Thus, untreated THP-1 cells had a typical spherical morphology and did not attach to the bottom of the culture plate wells (Figure 1e). After 24 h of PMA treatment (10 ng/mL), adhesion and partial cell spreading were observed, which was considered as the characteristic of M0 macrophages (Figure 1f). It is known that LPS induces the polarization of M0 macrophages into the M1 phenotype [32,34,35]. Therefore, to obtain proinflammatory M1 macrophages, M0 cells were treated with LPS for 72 h. As a result of this induction, all the cells were attached to the bottom of the culture plate and had an elongated morphology typical of M1 macrophages (Figure 1g), whereas THP-1 cells in the control group retained the morphology of a nonadherent culture.

Thus, the addition of NGF at a concentration of 100 ng/mL to PC12 cells led to their differentiation into neuron-like cells, as evidenced by the results of the morphological analysis. The THP-1 cell line was differentiated into macrophages via PMA and then treated with LPS to induce polarization toward the M1 phenotype.

### 3.2. Confirmation of the MSC Phenotype and Assessment of Preconditioning Effects

**Figure 2:**
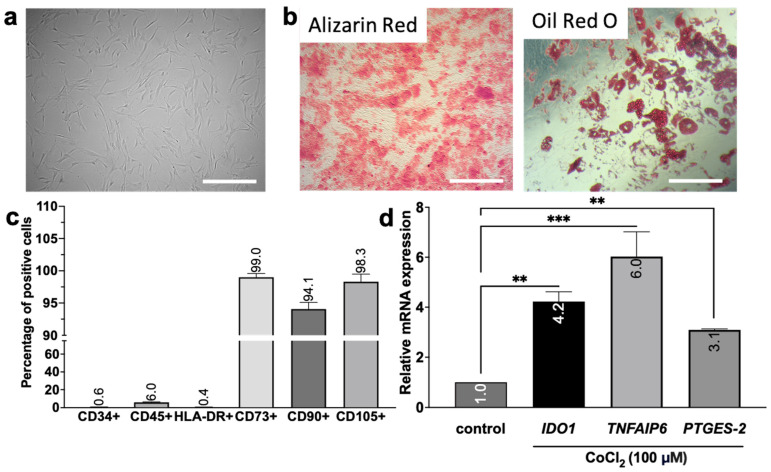
Phenotypic characterization and expression of immunosuppressive molecules in human adipose-derived MSCs. (**a**) The fibroblast-like morphology of the adhesive cells was examined via phase-contrast microscopy. (**b**) The multipotency of the cells was confirmed through the results of cytochemical staining for calcium deposition (Alizarin Red) and lipid accumulation (Oil Red O) after the induction of osteogenesis and adipogenesis, respectively. Scale bar, 100 μm. The immunophenotypic characteristics of the MSCs were investigated via flow cytometry. Negative expression of the CD34, CD45, and HLA-DR markers was observed, whereas positive expression of the CD73, CD90, and CD105 markers was detected (**c**). The relative mRNA expression of the *IDO1*, *TNFAIP6*, and *PTGES2* genes was also analyzed in hypoxia mimetic preconditioned MSCs (100 μM CoCl_2_ for 3 h) (**d**). Error bars indicate SEM. ** *p* < 0.01 and *** *p* < 0.001.

An investigation of MSC phenotypic characteristics was conducted. The cells had plastic adherence and a fibroblast-like (spindle-shaped) morphology, as shown in Figure 2a. After the cells were cultured in the medium that induced osteogenic differentiation, calcium deposits were observed via Alizarin Red staining (Figure 2b). Staining with Oil Red O also confirmed the occurrence of adipogenic differentiation (Figure 2b).

The MSC immunophenotype was analyzed via flow cytometry (Figure 2c). The cells were negative for the CD34 and CD45 markers and did not express the major histocompatibility complex class II antigen (HLA-DR). There was a weak positive reaction for CD73 and positive reactions for CD105 and CD90. These results were consistent with the MSC phenotype established by the International Society for Cellular and Gene Therapy (ISCT) [38].

Previously, we demonstrated that the activation of TLR3 results in an increase in the expression of MSC-specific immunosuppressive molecules [22]. In particular, treatment of MSCs with poly(I:C) induced the expression of *IDO1*, *TNFAIP6*, and *PTGES2*. The products of these genes are known to play a role in regulating the activation of immune system effector cells and contribute to the polarization of these cells into an anti-inflammatory phenotype [39,40,41,42,43]. For example, *IDO1* plays a role in the metabolism of tryptophan, a crucial substrate for T cells, and can be activated in MSCs during inflammatory conditions [44,45,46]. PGE2 and TSG-6 can inhibit T-cell proliferation and activation [39,41,47,48], as well as the polarization of macrophages toward the M1 phenotype, while promoting their polarization toward the M2 phenotype [42,43,49].

In this study, the expression of these specific molecules after the hypoxic preconditioning of MSCs was investigated (Figure 2d). Specifically, we pretreated MSCs with CoCl_2_, a hypoxia mimetic, for 3 h, at a concentration of 100 μM. This treatment led to increases in the expression of *IDO1*, *TNFAIP6*, and *PTGES2* of (4.2 ± 0.4)-fold, (6.0 ± 1.0)-fold, and (3.1 ± 0.1)-fold, respectively (Figure 2d). These results suggest that the proposed MSC preconditioning strategies increase the paracrine activity of cells and could be used to obtain enriched CMs.

### 3.3. MSC-Derived CMs Exhibit Neurotrophic Effects

**Figure 3:**
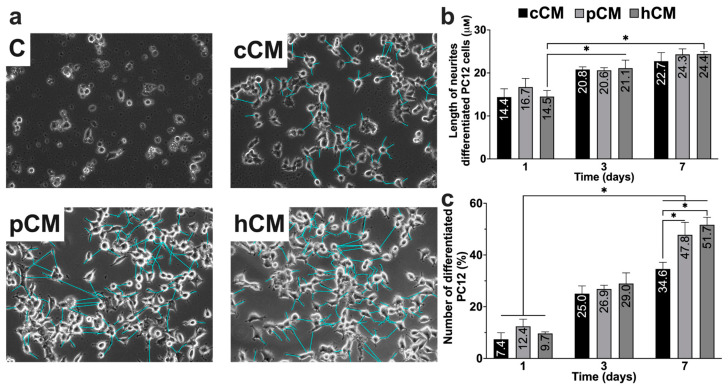
Investigation of the neurotrophic potential of CMs derived from MSCs. Morphology (**a**), neurite length (**b**), and number of differentiated PC12 cells (**c**) without treatment and after treatment with cCM, pCM, or hCM for 7 days. Scale bar, 50 μm. Neurites are highlighted in blue. Error bars indicate SEM. * *p* < 0.05.

MSCs exhibit neuroprotective properties, which are likely due to the secretion of factors that promote neurogenesis [50,51,52]. The neurotrophic potential of MSCs and MSC-derived CMs can be further improved by preconditioning the cells with poly(I:C) or CoCl_2_. To test this hypothesis, rat adrenal pheochromocytoma PC12 cells were treated with cCM, pCM, or hCM (Figure 3a). The differentiation of PC12 cells was assessed by counting the number of cells with neurites (Figure 3c) and measuring neurite length (Figure 3b) on the 1st, 3rd, and 7th days after adding CM to the PC12 cells.

The addition of various types of CMs resulted in the emergence and elongation of neurites in PC12 cells. After treating PC12 cells with CMs for 3 days, the length of the neurites ranged from 4 to 6 μm, whereas no neurites were detected in the control group (PC12 cells cultured in culture medium without CMs) (Figure 3a). On day 7 of differentiation, the mean length of the differentiated PC12 neurites was 22.7 ± 3.9 μm, 24.3 ± 2.6 μm, and 24.4 ± 1.2 μm after treatment with cCM, pCM, and hCM, respectively (Figure 3b), and there was no significant difference between the groups.

The treatment of PC12 cells with various types of CM led to an increase in the number of differentiated cells from day 1 to day 7 of exposure. Thus, treatment with cCM increased the number of differentiated cells by 27.2% (34.6 ± 5.1%), pCM increased the number of differentiated cells by 35.4% (47.8 ± 9.7%), and hCM increased it by 35.4% (51.7 ± 5.7%), compared with those in the control group on day 1 (7.4 ± 5.1%, 12.4 ± 5.5%, and 9.7 ± 1.1%, respectively). Notably, on day 7, the number of differentiated cells was significantly higher (*p* < 0.05) after induction with pCM or hCM than after induction with cCM (Figure 3a,c).

These results indicate that pretreatment with any type of MSC-derived CM induced the differentiation of PC12 cells into neuron-like cells, as indicated by their morphological characteristics. Additionally, the use of CMs derived from preconditioned MSCs appeared to yield a greater number of differentiated PC12 cells.

### 3.4. MSC-Derived CMs Exhibit Neuroprotective Properties

**Figure 4:**
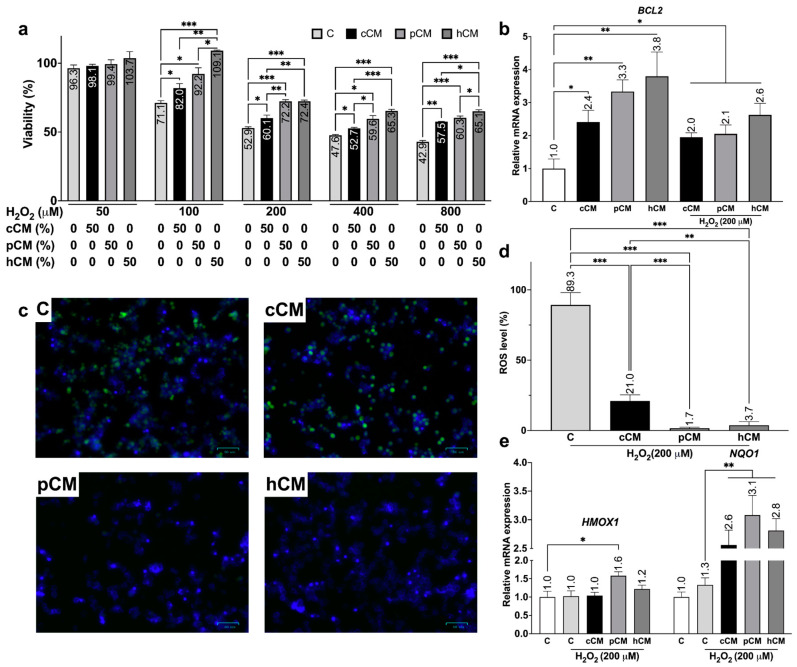
Study of the neuroprotective properties of CMs derived from MSCs. Evaluation of cell viability (**a**), the expression of the *BCL2* gene mRNA (**b**), the ROS level (**c**,**d**), and the mRNA expression of the *NQO1* and *HMOX1* genes (**e**) in differentiated PC12 cells without pretreatment (C, control group) and with pretreatment with different types of CM (cCM, pCM, and hCM) following H_2_O_2_-induced oxidative stress. Positive staining for DCF is shown in green, while cell nuclei are stained with Hoechst-33342 in blue. Scale bar, 50 µm (**c**). ROS production is expressed as a percentage (%) of DCF-stained cells relative to all cells (**d**). Error bars indicate SEM. * *p* < 0.05, ** *p* < 0.01, and *** *p* < 0.001.

MSCs secrete a variety of neuroprotective molecules [53,54,55]. Since pretreatment of MSCs enhances their paracrine activity, further investigation into the influence of different cell-preconditioning methods on the neuroprotective effects of their CMs in relation to a cellular model of ROS-induced neuronal injury was conducted (Figure 4).

The viability of dPC12 cells treated with H_2_O_2_ at concentrations ranging from 50 to 800 μM was analyzed 2 h after ROS induction via the MTT assay (Figure 4a). Exposure to a selected range of H_2_O_2_ concentrations led to a dose-dependent decrease in dPC12 cell viability. Pretreatment of dPC12 cells with all CM types resulted in an increase in the number of viable cells compared with that of the controls (untreated with CM) at high H_2_O_2_ concentrations (>200 μM, *p* < 0.05). In particular, cCM pretreatment increased PC12 cell viability by 7.2% (60.1 ± 4.5%) even at 200 μM H_2_O_2_ exposure, with pCM and hCM leading to increases of 19.3% (72.2 ± 2.5%) and 19.5% (72.4 ± 1.5%), respectively. These values were compared with those of the untreated control group of PC12 cells (52.8 ± 1.9%). Preconditioning MSCs with hypoxia mimetics appeared to be the most efficient method for enhancing the neuroprotective properties of CM. Therefore, the number of viable trPC12 cells was significantly higher (*p* < 0.05) than that in the control group (without treatment) or when other types of CM were used (100 and 800 μM H_2_O_2_).

It has been shown that the use of high concentrations of H_2_O_2_ and short exposure times can simulate acute short-term neurotoxicity [18,33]. Furthermore, *BCL2* expression typically correlates with increased cell survival [56,57,58]. Therefore, we evaluated the activation of the antiapoptotic mechanism in PC12 cells by measuring *BCL2* expression after treatment for 2 h with 200 μM H_2_O_2_ (Figure 4b). This concentration was selected on the basis of the results from the MTT assay (Figure 4a). Using qRT-PCR, it was demonstrated that pretreatment of dPC12 cells with cCM, pCM, or hCM promoted (2.4 ± 0.4)-fold, (3.3 ± 0.4)-fold, and (3.8 ± 0.7)-fold increases in *BCL2* expression, respectively, compared with the control (1.0 ± 0.3)-fold. Additionally, after CM pretreatment, 200 μM H_2_O_2_ was added to the dPC12 cells, and *BCL2* expression was measured again. We found that the change in *BCL2* expression was (2.0 ± 0.1)-, (2.1 ± 0.3)-, and (2.6 ± 0.3)-fold after pretreatment with cCM, pCM, and hCM, respectively (Figure 4b).

CMs derived from MSCs have antioxidant properties [7,59,60] and are involved in regulating the levels of ROS, which are associated with AD. In this study, the antioxidant effects of MSC-derived CMs were investigated using a specific substrate. CM-H_2_DCFDA (6-chloromethyl-2′,7′-dichlorodihydrofluorescein diacetate) does not fluoresce in the absence of ROS but is oxidized to fluorescing dichlorofluorescein (DCF) when ROS levels increase (Figure 4c). We measured the total fluorescence levels of control and pretreated dPC12 cells 2 h after the addition of H_2_O_2_ to assess the effects of CM. When dPC12 cells were treated with 200 μM H_2_O_2_, the cells fluoresced in the green spectrum, indicating substrate activation and active production of ROS (Figure 4c). The obtained values were normalized to the fluorescence level of intact cells (0%). Pretreatment with cCM, pCM, or hCM significantly (*p* < 0.001) reduced ROS levels in dPC12 cells (Figure 4c,d). The relative level of ROS during treatment with cCM was reduced to 21.0 ± 4.4% in dPC12, while treatment with pCM decreased it to 1.7 ± 0.7%, and treatment with hCM resulted in a 3.7 ± 2.6% decrease. The highest level of intracellular ROS was observed in the control dPC12 cells (89.3 ± 8.8%). Importantly, the levels of ROS in dPC12 differed significantly between different types of CM. Thus, the most pronounced antioxidant effect was observed as a result of pCM pretreatment (*p* < 0.01), while hCM also reduced ROS production more effectively than did cCM (*p* < 0.05).

To investigate the mechanism underlying the antioxidant effects of different CMs, PC12 cells were analyzed via qRT‒PCR after pretreatment with CMs and ROS induction (Figure 4e). The mRNA expression was normalized to the values for intact PC12 cells. The Nrf2/ARE pathway and its associated genes, including *NQO1* and *HMOX1*, are known to be activated in response to increased ROS levels to neutralize and normalize it. It was shown that pretreatment of dPC12 cells with different types of CM resulted in the activation of *NQO1* after the addition of H_2_O_2_ at a concentration of 200 μM (*p* < 0.01) (Figure 4e). Moreover, the relative mRNA expression of *HMOX1* tended to increase with hCM and was significantly altered after treatment with pCM compared with that in trPC12 cells without preincubation with CM (Figure 4e).

Thus, pretreatment of dPC12 cells with pCM and hCM resulted in a greater reduction in H_2_O_2_-induced intracellular ROS levels than did pretreatment with cCM. Moreover, exposure to hCM seems to lead to the observed effect on trPC12 cells due to the activation of *BCL2*, whereas in the case of pCM, it involves antioxidant-protective genes such as *NQO1* and *HMOX1*.

### 3.5. MSC-Derived CM Leads to Reduced LPS-Induced Polarization of M1 Macrophages

**Figure 5:**
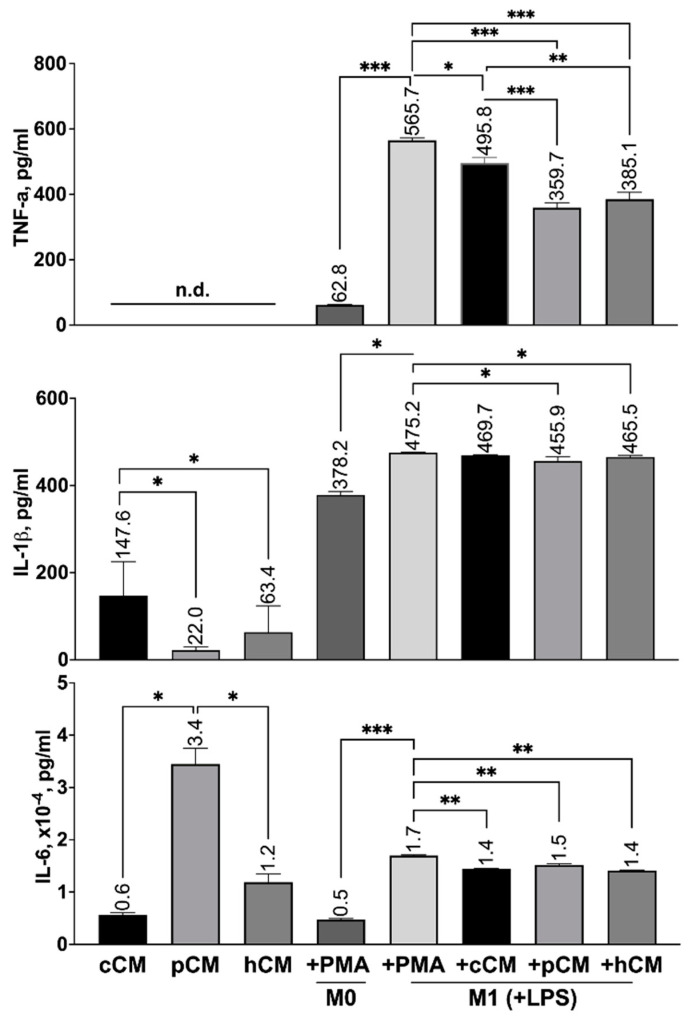
Effect of CM derived from MSCs on the LPS-induced differentiation of macrophages from the M0 phenotype to the M1 phenotype. The levels of the proinflammatory cytokines TNF-α, IL-1β, and IL-6 secreted by MSCs and macrophages in the M0 and M1 states. Error bars indicate SEM. n.d.—not detected. * *p* < 0.05, ** *p* < 0.01, and *** *p* < 0.001.

MSCs may have a paracrine-mediated effect on microglia/macrophages, including their polarization into a pro- or anti-inflammatory phenotype [49,61,62]. M0 macrophages were differentiated into the proinflammatory M1 phenotype by treatment with LPS at a concentration of 1 μg/mL for 72 h. The immunomodulatory potential of CMs was assessed by their effect on the differentiation of M0 cells into M1 macrophages. This was determined by measuring the levels of M1-specific proinflammatory cytokines in the conditioned medium via ELISA [63,64,65]. To represent the M1 phenotype, the following cytokines were chosen: TNF-α, IL-1β, and IL-6 (Figure 5).

TNF-α was not detected in cCM, pCM, or hCM, which may be due to the sensitivity of the ELISA kit used in this study (Figure 5). Compared with no treatment, treatment of M0 macrophages with 1 μg/mL LPS resulted in a significant increase in the TNF-α concentration in the conditioned medium (62.8 ± 1.6 pg/mL and 565.7 ± 12.6 pg/mL for M0 and M1 cells, respectively). A significant decrease in TNF-α levels was observed (*p* < 0.05 or *p* < 0.001) after the induction of differentiation in the presence of CM. M0-derived macrophages were treated with cCM, pCM, or hCM at TNF-α concentrations of 495.8 ± 19.2 pg/mL, 359.7 ± 15.5 pg/mL, or 385.1 ± 16.3 pg/mL, respectively.

IL-1β was detected in all types of MSC-derived CMs. Importantly, as a result of the pretreatment with either poly(I:C) or CoCl_2_, the levels of the proinflammatory cytokines in question were significantly reduced (*p* < 0.05). Additionally, untreated M0 macrophages constitutively produced IL-1β (378.2 ± 13.8 pg/mL), and treatment with LPS led to an increase in the concentration of IL-1β in the medium up to 475.2 ± 1.6 pg/mL. Treatment of cells with pCM or hCM resulted in a significant (*p* < 0.05) reduction in LPS-induced IL-1β secretion by M1 macrophages (455.9 ± 14.9 pg/mL and 465.5 ± 5.0 pg/mL, respectively).

In addition, MSCs constitutively secrete IL-6 (5677.7 ± 714.0 pg/mL). Both TLR3 activation and preconditioning with a hypoxia mimetic contributed to an increase in its production (34,489.9 ± 5231.0 pg/mL and 11,908.9 ± 2678.8 pg/mL for pCM and hCM, respectively) (Figure 5). It was also demonstrated that the induction of the M1 macrophage phenotype resulted in a significant increase in the IL-6 concentration (from 4724.5 ± 398.0 pg/mL for M0 to 16,990.6 ± 246.0 pg/mL for M1 macrophages). The addition of hCM and cCM to the IM containing LPS resulted in a significant reduction in IL-6 levels, from 16,990.0 ± 246.0 pg/mL to 14,433.4 ± 187.9 pg/mL and 14,082.7 ± 219.7 pg/mL for cCM and hCM, respectively. However, M1-characteristic IL-6 secretion was only slightly lower with pCM than with cCM and hCM (from 16,990.0 ± 246.0 pg/mL to 15,201.3 ± 354.8 pg/mL).

Therefore, the use of CM from preconditioned MSCs is a promising strategy for reducing the secretion of proinflammatory cytokines in LPS-stimulated macrophages.

### 3.6. MSC-Derived CMs Induce the Polarization of M0 Macrophages toward the M2 Phenotype

**Figure 6:**
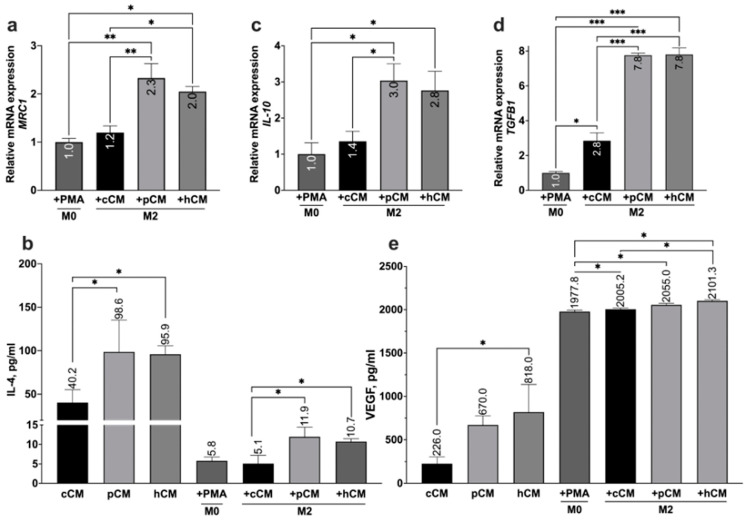
The effect of MSC-derived CM on the polarization of M0 macrophages toward the M2 phenotype. (**a**,**c**,**d**) Relative expression levels of *MRC1*, *IL-10*, and *TGFB1*, respectively. (**b**,**e**) Secretion levels of IL-4 and VEGF, respectively, following the induction of differentiation with CMs. Error bars indicate SEM. * *p* < 0.05, ** *p* < 0.01, and *** *p* < 0.001.

To investigate the effect of MSC-derived CMs on the differentiation of M0 macrophages into M2 macrophages, we treated M0 macrophages with cCM, pCM, or hCM for 72 h. M2 macrophages are known to play a role in reducing excessive inflammation by secreting anti-inflammatory cytokines [63,64,65,66]. In this study, the levels of IL-4 and VEGF in the culture medium of M2-induced macrophages were assessed via ELISA, as were the expression of genes that characterize the M2 phenotype (*MRC1*, *IL-10*, and *TGFB1*) via qRT‒PCR (Figure 6).

The maximum increase in the relative expression of *MRC1* was observed after the treatment of M0 macrophages with pCM, with a fold change of 2.3 ± 0.29. Compared with the control, treatment with hCM or cCM also increased *MRC1* expression by (2.0 ± 0.1)-fold and (1.2 ± 0.13)-fold, respectively (Figure 6a) (PC12 cells in culture medium). We showed that MSCs constitutively secreted IL-4 (40.2 ± 21.2 pg/mL) and that TLR3 activation and hypoxic preconditioning significantly increased (*p* < 0.05) the levels of this cytokine in pCM and hCM (98.6 ± 33.2 pg/mL and 95.9 ± 16.6 pg/mL, respectively) (Figure 6b). IL-4 was also detected in the CM from M0 macrophages (5.8 ± 1.36 pg/mL). Treatment of M0 cells with either pCM or hCM induced IL-4 secretion by M2-like macrophages (M2+pCM: 11.9 ± 4.2 pg/mL; M2+hCM: 10.7 ± 1.0 pg/mL) but not by those treated with cCM (M2+cCM: 5.8 ± 1.4 pg/mL).

All types of MSC-derived CM induced *IL-10* expression in M0 macrophages after treatment (Figure 6c). The maximum increase in *IL-10* expression was observed after incubation of M0 cells with pCM (fold change: 3.0 ± 0.3). Compared with untreated M0 cells, the cultivation of M0 macrophages in the presence of hCM led to an increase in *IL-10* expression by a factor of 2.8 ± 0.5. After incubation with cCM, *IL-10* expression further increased to 1.4 ± 0.3.

Treatment of M0 macrophages with all types of CM induced the expression of *TGFB1* (Figure 6d). Treatment with pCM or hCM resulted in (7.8 ± 0.11)-fold and (7.8 ± 0.37)-fold increases in the expression of *TGFB1*, respectively, whereas the use of cCM caused a (2.8 ± 0.45)-fold increase in *TGFB1* expression.

VEGF was detected in all types of MSC-derived CMs and in CM from M0 macrophages (Figure 6e). For cCM, the concentration of VEGF was 226.0 ± 134.9 pg/mL. Preconditioning MSCs with hypoxia mimetic and activating TLR3 increased VEGF secretion to 818.0 ± 258.5 pg/mL and 670.0 ± 147.7 pg/mL, respectively. For M0 macrophages, the level of VEGF secretion was 1977.8 ± 30.8 pg/mL. Compared with the treatment with cCM (2055. 0 ± 29.9 pg/mL) or pCM (2005.2 ± 23.5 pg/mL), the treatment of M0 macrophages with hCM resulted in the greatest increase in VEGF (2101.3 ± 21.9 pg/mL).

Therefore, the treatment of M0 macrophages with MSC-derived CMs contributes to their differentiation into the M2 phenotype on the basis of the increased expression and secretion of relevant M2 markers, such as *MRC1*, IL-4, *IL-10*, *TGFB1*, and VEGF. Moreover, differentiation into the M2 phenotype was more effective after treatment with CM from preconditioned MSCs than after treatment with CM from untreated MSCs.

## 4. Discussion

Key events in the pathogenesis of Alzheimer’s disease (AD) are associated with oxidative damage to neurons [67,68] and chronic microglial inflammation [67,69,70]. These processes can be studied via in vitro models, which are useful for the search and preliminary evaluation of therapeutic strategies aimed at reducing the progression of AD.

In this study, we investigated the effect of NGF on PC12 cells, a widely used cell line [32,33,71]. We found that after these cells were incubated with NGF for 7 days, they developed a characteristic neuronal-like morphology, similar to that reported in previous studies (Figure 1a–d) [33,71]. This finding is consistent with the results reported by Wiatrak et al. The researchers also observed morphological changes in PC12 cells toward sympathetic neurons and the formation of neuronal networks after NGF-induced differentiation [71]. To model oxidative damage to neurons, we initiated ROS formation by treating neuronal-like PC12 cells with H_2_O_2_. H_2_O_2_ is a well-known source of free radicals and can induce oxidative stress and apoptosis in cells [18,72,73]. This neurodamage model, however, is not sufficient for studying potential therapeutic agents in vitro, as it does not take into account the influence of these agents on the glial component of the brain [74,75,76]. Glial cells, such as microglia, are key cellular elements of the central nervous system, and they play important roles in maintaining contacts between nerve cells and in the development of neurodegenerative diseases, including AD [70,77,78]. Owing to ethical considerations, the study of human brain microglia has been limited. Therefore, the use of cellular models is a promising alternative [32,34]. In this study, THP-1 cells were differentiated into M0 macrophages (Figure 1f). These M0 cells are known to secrete and respond to cytokines similar to those of microglia in vivo, making them suitable for in vitro studies of microglial function [34]. Microglia become activated to the M1 phenotype in response to various stimuli, such as the direct toxic effects of amyloid beta oligomers and NFTs in Alzheimer’s disease [65,77]. We generated an M1 macrophage via LPS treatment to model this process (Figure 1g). Importantly, the differentiation of both PC12 and THP-1 cells plays a crucial role in the development of an AD model, as studies using intact cells have produced conflicting results [32]. In this study, we have developed a new experimental in vitro model of AD. This allowed us to investigate, for the first time, both the neuroprotective and anti-inflammatory effects of MSCs on induced neurodamage and microglial activation.

The use of MSCs and MSC-derived CMs is a promising approach for the treatment of neurodegenerative diseases [12,19,53,79,80], including AD [26,81,82,83,84]. In this study, we investigated the immunosuppressive, neurotrophic, antioxidant, and antiapoptotic properties of the MSC secretome in an obtained in vitro model of AD. The conditioned medium derived from untreated MSCs (cCM) had neuroprotective effects, as expected [61,80,81,82]. Pretreatment of dPC12 cells with cCM activated the Nrf2/ARE antioxidant pathway, as confirmed by increased expression of Nrf2-controlled genes, such as *NQO1* (Figure 4e). In addition, cCM treatment influenced the M1/M2 balance of model microglia by promoting the production and expression of anti-inflammatory molecules and reducing the secretion of proinflammatory cytokines (Figure 5 and Figure 6a–d). These findings are in good agreement with those of previous studies. For example, it has been reported that MSC-derived CM and exosomes have neuroprotective effects, which are associated with the activation of the Nrf2/ARE pathway in neurons [85,86,87] and the secretion of immunosuppressive cytokines [14,16,43,80,88]. In addition, neuroprotection may be mediated by neuroregulatory molecules in CM from MSCs, such as NGF, BDNF, and GDNF [52,61,89,90]. In this work, we observed the induction of PC12 cell differentiation into neuron-like cells using CM from MSCs (Figure 3a–c). This process is likely mediated by the production of neurotrophic factors, as evidenced by our previous proteomic profiling results [22], which revealed that intact MSCs express MANF (Mesencephalic Astrocyte-Derived Neurotrophic Factor), NPTN (Neuroplastin), ASHNAK, and PACSIN (Protein Kinase C and Casein Kinase Substrate in Neurons). These proteins are known to be involved in neuronal development and function.

Strategies for enhancing the therapeutic efficacy of MSC-derived CM have been actively discussed in recent years [20,23,42,54]. Pretreatment of MSCs with proinflammatory factors leads to the intensification of their paracrine activity and immunosuppressive properties [20,23,42,91,92]. We previously showed that MSCs express functional TLR3, and TLR3’s activation results in the increased expression of anti-inflammatory molecules (*IDO1*, *TNFAIP6*, *PTGES2*, etc.) [22]. Hypoxia can also lead to the enrichment of the MSC secretome [25,26,29,53], making the use of hypoxia mimetics a promising strategy. In this study, we investigated the effects of preconditioning MSCs with CoCl_2_, a hypoxia mimetic, on the expression of immunomodulatory factors and the secretion of angiogenic factors (Figure 5 and Figure 6). We showed, for the first time, that the incubation of MSCs with cobalt chloride promoted the increased expression of *IDO1*, *TNFAIP6*, and *PTGES2* (Figure 2d). These factors play important roles in the immunomodulatory effects of MSCs [39,44,53,93,94,95]. Next, we investigated the effects of TLR3 activation and hypoxia mimetic preconditioning on the neurotrophic and neuroprotective properties of MSC-derived CM. Indeed, PC12 cells differentiated into neuronal-like cells more effectively after treatment with hCM than after treatment with cCM or pCM. In particular, hCM treatment led to the greatest number of cells with neurites after 7 days (Figure 3c), probably due to the higher concentration of VEGF in hCM than in hCM and pCM (Figure 6e). Thus, it has been previously reported that hypoxic preconditioning of MSCs can enhance the neurotrophic properties of CM due to the secretion of vascular endothelial growth factor (VEGF) [52,90,96] and other molecules [52,61,89,90]. However, Zayed et al. did not find any effect of hypoxic preconditioning on the neurotrophic properties of MSCs. These contradictory results may be due to differences in the preactivation protocols used, the source of the stem cells, or the model of cells used [97]. We did not observe significant differences in the efficiency of PC12 cell differentiation upon exposure to pCM or cCM. According to previously published proteomic data, TLR3 activation does not lead to the induction of neurotrophic factor expression [22], and as a result, its concentration in pCM does not differ from that in cCM.

Next, we assessed the effect of MSC preconditioning on the neuroprotective properties of CM. We found that the viability of PC12 cells treated with pCM or hCM before exposure to H_2_O_2_ was higher (*p* < 0.05) than that of PC12 cells treated with cCM or without treatment (Figure 4a). These results are likely due to the activation and increased expression of the antiapoptotic protein BCL2 in PC12 cells following pCM and hCM treatment (Figure 4b). Previous studies have demonstrated that the induction of BCL2 expression in PC12 cells contributes to their resistance to apoptosis following H_2_O_2_ treatment [56]. The enhancement of the neuroprotective effects of CM from hypoxically preconditioned MSCs may be associated with the activation of the phosphatidylinositol 3-kinase (PI3K)/protein kinase B (AKT) pathway [55,98,99,100]. This pathway and HIF-1α are known to result in the increased production of regenerative and neurotrophic factors, such as VEGF, FGF, BDNF, and NGF, in MSCs [55,60,101]. The neuroprotective effects observed after pCM pretreatment may also be associated with galectin-3 activation, which has been shown to reduce neurotoxicity in vitro [22,102] and is induced in MSCs after TLR3 activation [22]. It has been reported that TLR3 activation can also induce HIF-1α, as shown in a murine bone marrow-derived macrophage model [103]. This may be the reason for similar results when comparing the neuroprotective effects of pCM and hCM. However, the exact mechanism by which hypoxia mimetic treatment and TLR3 activation in MSCs led to the enhanced neuroprotective properties of conditioned medium is yet to be investigated.

Next, we demonstrated that pretreatment of PC12 cells with hCM or pCM, but not cCM, led to decreased levels of H_2_O_2_-induced reactive oxygen species (ROS) (Figure 4c, d). Specifically, the level of ROS was lower in pCM than in cCM and hCM, likely due to the highest activation of genes involved in the oxidative-stress response, such as *NQO1* and *HMOX1* (Figure 4e). A similar mechanism was observed in a study of the secretome of neuronal and glial progenitor cells derived from induced pluripotent stem cells (iPSCs), which also reduced glutamate-induced oxidative stress in PC12 cells [104]. Importantly, in AD diagnosis, the levels of corresponding proteins (NQO1 and HO-1) in neuronal cells are significantly reduced, and activation of the Nrf2/ARE signaling pathway is impaired [85,105,106,107]. It has also been reported that inhibitors of the NQO1 and HO-1 proteins reduce the effectiveness of neuroprotective agents, including by increasing levels of ROS [108]. While modulation of the HO-1 system plays an important role in the development of Alzheimer’s disease [107], activation of the NQO1 protein is critical for reducing apoptosis in PC12 cells [109]. Specifically, the level of NQO1 production can be correlated with the progression of AD due to gene expression and alternative splicing of proteins involved in apoptosis [109]. Therefore, the use of pCM and hCM may be a potentially effective strategy to reduce ROS levels in damaged neurons.

We investigated the effects of MSC-derived CM on the differentiation of macrophages into pro- and anti-inflammatory phenotypes. The formation of Aβ oligomers and their binding to microglia induces their activation in AD, as previously reported [70,77,110]. The use of CM from preconditioned MSCs was more effective in reducing LPS-induced M1 macrophage activation compared to cCM (Figure 5). We specifically investigated the effects of CM on the secretion of the proinflammatory cytokines IL-1β and TNF-α. These factors are involved in the pathogenesis of AD and the maintenance of chronic inflammation, as reported in previous studies [64,77,111,112]. In addition, the proportion of M1-differentiated macrophages reportedly correlates with the level of production of certain cytokines [113,114]. Our study revealed that treatment with pCM most effectively reduces M1 microglial activation. Previous studies have also found that TLR3 activation in cord blood-derived MSCs enhances the anti-inflammatory effects of their vesicles on RAW 264.7 macrophage activation induced by LPS [115]. The mechanism by which MSCs participate in the regulation of macrophage M1 polarization has been reported to be mediated by various microRNAs (miRNAs), including miR-21, miR-320a, miR-423, miR-100, and miR-26a. These miRNAs target genes involved in inflammation and immune response pathways, such as PTEN, NLRP3, mTOR, and TLR3 [116]. We also assessed IL-6 levels in CM from MSCs and macrophages. IL-6 is a cytokine that is known to mediate proinflammatory responses [117,118], but it has also been reported to play a role in the regulation of neuroinflammation [115,117,119] and its effects on the brain [18,120,121]. Thus, Lin et al. showed that IL-6 can have a direct effect on microglia under conditions of neuroinflammation by stimulating the phosphorylation of STAT3, STAT1, and ERK and also modulating inflammation, depending on the microenvironment, such as the presence of IFN-γ [122]. We have shown that IL-6 is secreted by untreated MSCs and is induced by TLR3 activation and treatment with a hypoxia mimetic (Figure 5). The activation of M0 macrophages with LPS also leads to increased secretion of this cytokine (Figure 5). As a result of treating activated macrophages with pCM and hCM, the level of IL-6 in the CM was reduced equally effectively and was comparable to that resulting from the use of cCM. Zhang et al. previously showed that MSCs exert a neuroprotective effect on PC12 cells by regulating the microenvironment disrupted by H_2_O_2_, specifically due to the production of the cytokines IL-6 and IL-10 [18]. In addition, it has been reported that IL-6 produced by MSCs plays a role in neuroprotection by reducing autophagy in hippocampal neurons, partly through the AMPK/mTOR pathway [121]. We have noted a decrease in the secretion of IL-6 by activated macrophages as a result of incubation with MSC-derived CM in our work, which could potentially be seen as example of the immunosuppressive properties of these CMs. However, our findings also suggest a dual role for IL-6 in processes associated with neuroinflammation [117,118,120,122]. It has been suggested that promoting the polarization of microglia toward the anti-inflammatory M2 phenotype could be a potential strategy for treating AD [65]. In this study, we investigated the effect of treating M0 macrophages with CM from preconditioned MSCs on their differentiation toward the M2 phenotype (Figure 5). M2 macrophages are induced by Th2 cytokines, such as IL-4, IL-10, and TGF-β [64]. These cells also secrete VEGF [64,123,124,125,126] and express *MRC1* [123]. To confirm M2 polarization in our study, we examined the expression of these markers. For the first time, we showed that TLR3 activation in MSCs or treatment with CoCl_2_ leads to an increase in IL-4 levels in the CM (Figure 6b). It is known that IL-4 not only serves as a marker and inducer of M2 microglia differentiation but also has a neurotrophic effect [63], is involved in reducing Aβ levels in the brain [127], and can be used as a neuroprotector [128]. In addition, we reported that treatment of M0 macrophages with pCM or hCM, but not with cCM, increases the expression of *MRC1* and *IL-10* and the secretion of TGF-β and VEGF (Figure 6a,c–e). Thus, pCM and hCM appear to modulate the differentiation of M2-like microglia, likely due to the increased concentration of IL-4 in these CMs. As previously shown, IL-4-overexpressing MSCs can modulate MRC1 expression and/or secretion by macrophages both in vitro [124] and in vivo [123]. We also observed increases in the expression of *IL-10* and *TGF-β* in macrophages after treatment with CM from preconditioned MSCs. This can be seen as an immunosuppressive effect of CM and the polarization of macrophages toward the M2 phenotype [19,49,64,125]. In particular, IL-10 and TGF-β are reported to mediate the therapeutic effects of MSCs in inflammatory conditions by reducing the activation of microglia and astrocytes [125]. Additionally, IL-10 has been shown to act as a neuroprotector, as IL-10 secretion promotes neurogenesis in the hippocampus of mice with Alzheimer’s disease [49].

In addition, preconditioning MSCs with CoCl_2_ resulted in the greatest induction of VEGF, as expected (Figure 6e) [29,129,130]. Treatment with hCM also had the greatest effect on VEGF production by macrophages. VEGF has been reported to modulate the polarization of macrophages from M0 to M2 [131,132]. The inhibition of M2 differentiation is accompanied by a decrease in VEGF expression [133]. Interestingly, IL-10 can increase VEGF secretion in M2 macrophages, leading to the inhibition of M1 polarization [134]. Most likely, hCM also influences macrophage differentiation through the high secretion of neurotrophic factors, such as GDNF. Previous studies have shown that GDNF, secreted by MSCs, regulates the balance between M1 and M2 microglia and stimulates M2 differentiation through the PI3K/AKT signaling pathway [89]. The use of CM from preconditioned MSCs results in increased expression and secretion of *MRC1*, *IL-10*, *TGFB1*, and IL-4 in macrophages (Figure 6a–d). The efficiency levels of pCM and hCM do not differ significantly from each other but are higher than that of cCM. In addition, the increased concentrations of IL-6 and IL-4 in pCM likely mediate the neuroprotective effects observed in PC12 cells treated with H_2_O_2_. For hCM, neuroprotection during induced neurodamage is likely associated with the activation of the PI3K/AKT pathway and the secretion of growth factors such as VEGF and GDNF. The enhancement of macrophage polarization toward the M2 phenotype and the reduction in LPS-induced M1 differentiation following treatment with pCM and hCM may also be associated with the upregulation of immunosuppressive molecules such as *IDO1*, *TNFAIP6*, and *PTGES2* in MSCs [135]. We have previously demonstrated that these molecules are involved in regulating T-cell activation and proliferation in MSCs after TLR3 stimulation and subsequent coculture [22]. In this study, we reported that hypoxic preconditioning also increases the expression of these genes in MSCs, which are known to play a role in modulating the immune response and microglial polarization [42,43,53,62]. Increased IDO production has been reported to lead to the transformation of monocytes into IL-10-secreting CD206+ M2-like macrophages [42]. Additionally, high TSG-6 expression in MSCs decreases the level of LPS-induced M1 macrophage polarization and promotes microglia polarization toward the M2 phenotype [62]. The role of PGE2 secreted by MSCs in regulating macrophage/microglial activation and its potential effect on M2 macrophage differentiation has also been documented [43]. Notably, PGE2-stimulated M2 macrophages exhibit high levels of VEGF secretion [134], which aligns with our findings for pCM. The effects of pCM and hCM on the balance between M1 and M2 macrophages are likely similar, as they both activate anti-inflammatory molecules. However, the degree of induction of these molecules differs, which could influence the intensity of the effect of these conditioned media.

## 5. Conclusions

MSC preconditioning may be a promising strategy for enhancing the neuroprotective and immunosuppressive activity of these cells. Specifically, TLR3 activation in MSCs contributes to more potent immunosuppressive effects of CM against proinflammatory M1 macrophages and may prevent the development of neuroinflammation by maintaining a balance between the M1 and M2 microglial phenotypes. Additionally, conditioned medium (CM) derived from hypoxia mimetic-preconditioned MSCs showed increased neurotrophic effects due to activation of the antiapoptotic response following H_2_O_2_-induced injury to neuronal cells in vitro. For the first time, CM from MSCs preconditioned with a TLR3 ligand or the hypoxia mimetic CoCl_2_ has been shown to have enhanced neuroprotective properties in an in vitro model of Alzheimer’s disease compared with CM derived from untreated MSCs. These findings are of interest for the further development of CM from preconditioned MSCs for Alzheimer’s disease therapy. However, a more in-depth study of the mechanisms by which these CMs exert neuroprotective effects is needed.

## Figures and Tables

**Table 1:** Components of the induction medium used for MSC differentiation (Sigma-Aldrich, USA).

Culture Medium	Components	Concentration
Osteogenic	Dexamethasone	100 nM
	β-Glycerol phosphate	10 mM
	Ascorbate-2-phosphate	0.5 mM
Adipogenic	Insulin	1 μg/mL
	Isobutyl-1-methylxanthine	0.5 mM
	Dexamethasone	0.5 μM

**Table 2:** Sequences of the gene primers.

Cell Types	Genes	Forward Sequences	Reverse Sequences
MSCs	*ACTB*	TCAGAAGGATTCCTATGTGGGCGA	CACGCAGCTCATTGTAGAAGGTGT
	*GAPDH*	TCGACAGTCAGCCGCATCTTCTTT	ACCAAATCCGTTGACTCCGACCTT
	*IDO1*	CCCTTCAAGTGTTTCACCAAATC	GTCTTCCCAGAACCCTTCATAC
	*TNFAIP6*	AAGATGGGATGCCTATTGCTAC	ATTTGGGAAGCCTGGAGATTTA
	*PTGES2*	CAGCACTTCACGCATCAGTT	GTCTAGCCAGAGTTTCACCGTA
THP-1	*MRC1*	GCAAAGTGGATTACGTGTCTTG	CTGTTATGTCGCTGGCAAATG
	*IL10*	TCAGGCTGAGGCTACGG	AGATGTCAAACTCACTCATGGC
	*TGFB1*	CGTGGAGCTGTACCAGAAATAC	CACAACTCCGGTGACATCAA
PC12	*GAPDH*	CCATCAACGACCCCTTCATT	GACCAGCTTCCCATTCTCAG
	*HMOX1*	CCAACATTGCCGTGCCAC	GCTCCTGCAACTCCTCAAAGAG
	*NQO1*	TGCAGCGGCTTTGAAGAAGA	AGGGTCCTTCAGTTTACCTGTG
	*BCL2*	GATGACTGAGTACCTGAACCG	CAGAGACAGCCAGGAGAAATC

## Data Availability

All data associated with this study are available in the main text or are available through the corresponding author upon request, due to privacy.
